# Grasping affordance judgments depend on the object emotional value

**DOI:** 10.3389/fnhum.2024.1331253

**Published:** 2024-03-18

**Authors:** Matheus Ribeiro Felippin, Ivo Lopes Azevedo, Ghislain Saunier, Les Keniston, Anaelli Aparecida Nogueira-Campos

**Affiliations:** ^1^Laboratory of Cognitive Neurophysiology, Institute of Biological Sciences, Department of Biophysics and Physiology, Federal University of Juiz de Fora (UFJF), Juiz de Fora, Minas Gerais, Brazil; ^2^Post-Graduation Program in Biological Sciences, Carlos Chagas Filho Institute of Biophysics, Federal University of Rio de Janeiro (UFRJ), Rio de Janeiro, Brazil; ^3^Laboratory of Motor Cognition, Department of Anatomy, Federal University of Pará (UFPA), Belém, Brazil; ^4^Post-Graduation Program in Human Movement Sciences, Federal University of Pará (UFPA), Belém, Brazil; ^5^Department of Biomedical Sciences, Kentucky College of Osteopathic Medicine, University of Pikeville, Pikeville, KY, United States; ^6^Post-Graduation Program in Rehabilitation Sciences and Physical-Functional Performance, Federal University of Juiz de Fora (UFJF), Juiz de Fora, Minas Gerais, Brazil

**Keywords:** affordance, grasping, emotion, object size, visual perception, visuo-motor integration, motor planning, valence

## Abstract

**Introduction:**

The concept of affordance refers to the opportunities for action provided by the environment, often conveyed through visual information. It has been applied to explain visuomotor processing and movement planning. As emotion modulates both visual perception and the motor system, it is reasonable to ask whether emotion can influence affordance judgments. If present, this relationship can have important ontological implications for affordances. Thus, we investigated whether the emotional value of manipulable objects affected the judgment of the appropriate grasping that could be used to interact with them (i.e., their affordance).

**Methods:**

Volunteers were instructed to use a numerical scale to report their judgment on how an observed object should be grasped. We compared these judgments across emotional categories of objects (pleasant, unpleasant and neutral), while also considering the expected effect of object size.

**Results:**

We found that unpleasant objects were rated as more appropriately graspable by a precision grip than pleasant and neutral objects. Simultaneously, smaller object size also favored this judgment. This effect was seen in all emotional categories examined in equal magnitude.

**Discussion:**

Our findings suggest that the emotional value of objects modulates affordance judgments in a way that favors careful manipulation and minimal physical contact with aversive stimuli. Finally, we discuss how this affective aspect of our experience of objects overlaps with what affordances are conceptualized to be, calling for further reexamination of the relationship between affordances and emotions.

## 1 Introduction

### 1.1 What are affordances?

It is already well settled that looking at objects not only causes a perception of their physical properties, but automatically evokes in the observer potential actions that they could perform on and with these objects. This set of possible actions was called “affordances” by Gibson (1979). He proposed that affordance perception depends on the morphological and biomechanical compatibility between the agent and target of the action and occurs automatically: independently of the observer’s intention to interact with the observed object. His concept of affordance is an important part of his theory of direct visual perception, which is part of a larger theoretical framework of ecological psychology, developed by Gibson and other psychologists as a non-representational alternative to more traditional cognitivist views on the study of perception-action (Lobo et al., 2018). Nevertheless, the concept of affordance has been widely used in psychology and neuroscience to understand, among other topics, visuomotor processing, tool use and movement planning/control, and is often employed disassociated from ecological psychology approaches or even embedded in representational theoretical frameworks (de Wit et al., 2017; Osiurak et al., 2017).

One reason for this might be that even if we do not assume the whole theories and approaches proposed by ecological psychologists, the notion that the environment automatically evokes potential actions in animals, conveyed by key elements of visual information that can be perceived directly (or with a lesser dependence on “mental representations” or other cognitive intermediaries), is still an intriguing and powerful idea. And as we will describe ahead, this idea has been scientifically fruitful, even in more representational, neurocognitivist approaches. In this paper we use a modern definition of affordance, as proposed by Osiurak et al. (2017):

*“An affordance is an animal-relative, biomechanical property specifying an action possibility within a body/hand-centered frame of reference. Affordances correspond to a description of this possibility at a physical, but not at a neurocognitive level. At the neurocognitive level, the issue is to understand how an animal can perceive affordances* (i.e.*, affordance perception*)*.”*

This definition is very similar to that of Gibson, in that, for both, affordances are descriptions of interaction possibilities, like “grasping a hammer using the whole hand,” “pinching a coin with two fingers” or “stepping on top of a rigid surface” for example. Nevertheless, Gibson considered the possible interactions between tools and their target objects to also be affordances (which would also be directly perceived by animals), whereas Osiurak et al. (and we herein) use the term to refer only to possible actions necessarily involving the body of animals, as meant by “body/hand-centered frame of reference.” For example, Gibson states (1979):

*“A rigid staff also affords leverage, and that use is a lever. A pointed elongated object affords piercing – if large it is a spear, if small a needle or awl […] A rigid object with a sharp dihedral angle, an edge, affords cutting and scraping; it is a knife.”*

Note that these descriptions of interactions do not depend on the biomechanical compatibility between animal and tool, but instead on the compatibility between tool and the inanimate target object. They do reveal, however, that there is an expectation of a specific, appropriate way to use tools (even though other actions or uses are possible), which is reflected in both concepts of affordances presented thus far. More generally, like Gibson, many affordance definitions and theories of action planning and motor control rely heavily on this notion of an appropriate, natural, best for the context or more intuitive action for each specific object.

Yet, objects can almost always be interacted with in many ways, and it can often be argued that more than one of these are viable or stable movements to be used. Furthermore, even the movement judged to be the best form of interaction can be different depending on the context. For example, a screwdriver could be interacted with a “whole-hand grasp,” in which all fingers close together around the object and against the thumb and palm; but it could also be grasped with a finer grip, applying pressure through the fingertips in a claw-like posture. These are both functional manipulations and we can easily imagine situations in which one would be better than the other (for instance, if there are obstacles limiting wrist movements). Likewise, we can imagine how different screwdrivers with different weights, widths and other features could favor one movement over the other in this judgment. Furthermore, small changes in movement parameters (like grip force, points of contact, joint angles) could probably be successfully realized without meaningful differences in actual task performance. Are these different “movement settings” different enough to be classified as different “movements” or “actions”? If so, then we are talking not of two but of an infinite (or arbitrary) number of movements.

It seems that, according to our definition, objects “have” not one but multiple affordances because multiple interactions with them can be described in the same context of animal-environment relationships. But if we were to adopt this understanding and use of the term, “affordance” would mean very much the same as “action possibility” and would lose its uniqueness and theoretical usefulness as a specific concept in the study of action-perception. Therefore, to explicitly convey this notion of “*the* appropriate interaction among other action possibilities,” we will use the term “affordance judgment” to mean the personal judgment (at the level of conscious subjective experience) of what is *the* most appropriate movement for interacting with an object. The definition of Osiurak et al. (2017) already points at this when it states that an affordance is a property “specifying an action possibility,” but with “judgment” we want to specifically emphasize this evaluation of appropriateness.

### 1.2 Affordance perception and judgment

A common idea in the study of visually guided behavior is that there must be a sensory-motor system capable of automatically processing visual features of objects into motor plans that could be readily used to perform actions. Many neurophysiological studies support such idea. Electrophysiological recordings performed within monkey premotor area F5 described a class of bimodal visuomotor neurons, namely “canonical neurons” (Rizzolatti and Fadiga, 1998), that become active when monkeys execute a goal-directed action or passively observe an object (Murata et al., 1997; Rizzolatti and Arbib, 1998). It is suggested that these cells may be part of a system for detecting specific object features and processing them into action-specific information (Rizzolatti and Arbib, 1998; Murata et al., 2000). These neurons, together with another class of visuomotor neurons called “mirror cells” (di Pellegrino et al., 1992) and with other areas (such as the anterior intraparietal region, posterior parietal cortex and premotor cortex), are proposed to be all part of a network encompassing the parietal and frontal cortices which processes the visual features of the object into specific motor actions for interacting with it (Gentilucci et al., 1988; Jeannerod et al., 1995; Matelli and Luppino, 2000; Rizzolatti and Luppino, 2001; Dum and Strick, 2005; Brochier and Umiltà, 2007).

Consistent with studies in monkeys, behavioral and neurophysiological studies in humans have also shown that the passive observation of objects can generate electrophysiological changes in the human motor cortex, thus facilitating actions for interacting with these objects. For example, Makris et al. (2011) designed an experiment where participants were asked to rapidly perform a precision or power grip in response to changes in the background color of a screen, while graspable and non-graspable objects were presented on the screen (unrelated to the task, but potentially congruent with the required grasping response). They found that when these objects elicited a type of grasping congruent with that required by the task, participants’ reaction times were significantly faster. They suggested that the observation of objects was capable of automatically priming a motor action for interacting with them, even if they were unrelated with the actual task. Indeed, in a second experiment, these authors showed that passive observation of those objects tuned the corticospinal excitability in an affordance-specific manner (Makris et al., 2011). Similar findings regarding behavioral and neurophysiological effects associated with object viewing have also been shown by others (Tucker and Ellis, 1998, 2001; Craighero et al., 1999; Ellis and Tucker, 2000; Buccino et al., 2009; Proverbio et al., 2011; Franca et al., 2012).

### 1.3 Emotion influences visual perception

In another line of evidence, the visual perception of certain object features and their spatial relationships with the observer has been shown to be modulated by emotion. Notably, unpleasantness can elicit perception changes that accentuate aversive stimuli features such as slant, size, closeness, and duration (Stefanucci et al., 2008; Van Ulzen et al., 2008; Smith et al., 2011; Cole et al., 2013). These studies show that emotions can favor withdrawal reactions, high alertness and attention, careful manipulation and minimal physical contact with possible sources of threat. For instance, Van Ulzen et al. (2008) showed that circles were judged as bigger when containing unpleasant rather than pleasant or neutral stimuli. This overestimation may have been evolutionarily useful in emphasizing the importance of aversive stimulus. By bringing attention to a potential threat and even suggesting that it is closer to the observer than in reality, such emotional modulations in perceptions may better allow for adaptive behavior (e.g., fleeing posture). Cole et al. (2013) corroborated this hypothesis: first, they found that participants’ estimates of distance to a live spider were correlated with their reported levels of feeling threatened by it. Finally, they manipulated participants’ judgment of an actor to be either threatening, disgusting or neutral and found that participants also estimated to be closer when they felt threatened. Conversely, Balcetis and Dunning (2010) investigated if the desirability of certain stimuli might influence how close they are perceived to be. In a series of five experiments, the authors showed that participants tended to see desirable objects as physically closer than less desirable objects, and this biased distance perception was revealed both through verbal reports as well as through behavioral data on actions toward objects and visual matching tasks. Analogous with what has been discussed about threatening stimuli, the authors of this study also suggest that these effects elicited by the stimulus emotional value imply a behavioral function of priming proper interactions within a specific affective context.

### 1.4 Can emotion influence affordance judgment?

For all the evidence presented above, we deemed it reasonable that the processes underlying affordance judgment might be influenced by the emotional value “of” objects, i.e., the emotional value typically assigned to them in a neutral setting (without explicit influence of other extrinsic emotional stimuli or specific affective contexts). Thus, we sought to evaluate whether the emotional category of an object (pleasant, unpleasant or neutral) can affect the judgment of the appropriate grasp to interact with it. Indeed, recent evidence suggests that the emotional valence of an object can modulate: (1) kinematic features of grasping actions (Esteves et al., 2016), (2) corticospinal excitability during motor preparation and passive observation of grasping actions (Nogueira-Campos et al., 2014, 2016) and (3) action predictions of grasping movements of other people (Saunier et al., 2023). Therefore, we wished to expand upon this line of research by explicitly assessing whether emotional valence might affect affordance judgments, which could provide an explanation for such findings.

In the real world, objects can be manipulated with many possible movements, but given a specific object in a specific context, one or some of these movements will inevitably be considered more appropriate than others for whatever goals, criteria or other considerations imposed by the situation. The ability to make such judgments by only looking at an object (independent of any intention to interact with it) is what we call affordance judgment, and here we analyze it using a paradigm of contrasting two prototypical grasping movements: an index-finger and thumb “precision grip” and a whole-hand “power grip.” These movements have been thoroughly studied (see Castiello, 2005 for a review), with detailed descriptions on how their kinematic profiles are strongly dependent on object properties (Jeannerod, 1984; Paulignan et al., 1991, 1997; Smeets and Brenner, 1999), especially size, which exhibit strong correlations with key kinematic parameters, such as maximum grip aperture (Santello and Soechting, 1998; Meulenbroek et al., 2001; Cuijpers et al., 2004; Domalain et al., 2008; Thakur et al., 2008; Jarque-Bou et al., 2016). Specifically, these movements are useful for our investigation on affordance judgment because: (1) they have very similar kinematic profiles, in that they are both pinching movements mainly involving arching of the hand and fingers, thumb opposition and applying a constriction force (Thakur et al., 2008; Jarque-Bou et al., 2016); (2) they can be used to manipulate objects in a similar way (for instance, grasping a cylindrical-shaped object to move it to another location) and (3) each can be considered more fitting for interacting with some objects rather than others, depending on their features. For example, thick objects tend to be grasped by a power grasp (Jarque-Bou et al., 2016), but this preference significantly depends on the orientation of the object when it is asymmetrical (Cuijpers et al., 2004). Analogously, here we investigate whether emotion can influence affordance judgment when precision and power grasps are presented as opposite poles on a continuum of action possibilities for object manipulation.

Our hypothesis was that affordance judgment would be influenced by the emotional value of objects, which would be confirmed if we observed a difference of affordance scores between the neutral objects and the emotionally-laden ones (pleasant and unpleasant). Conversely, this hypothesis would be rejected if there were no difference across all the emotional categories of objects.

## 2 Materials and methods

### 2.1 Participants

Thirty-one adult volunteers took part in this experiment (14 women, 17 men, mean age 26.1 ± 7.4 and 25.2 ± 8.5, respectively). One of them was left-handed and all others were right-handed as assessed by the Edinburgh Handedness Inventory (Oldfield, 1971). All participants had normal or corrected to normal visual acuity. Participants were blind to the experiment design and actual purpose, knowing only that we sought to study how the motor system processes visual information. This study was approved by the local Ethics Committee (CAAE: 64960417.8.0000.5147) and complied with the Declaration of Helsinki as 2013. All volunteers gave informed consent before participating in the experiment.

### 2.2 Stimuli

We assembled a stimulus set composed of 102 pictures of manipulable, emotion-laden and ecologically relevant objects with varying shapes and sizes.^1^ These objects were previously classified (Azevedo et al., 2021) by means of the Self-Assessment Manikin Scale (Bradley and Lang, 1994) as either *pleasant* (21 objects)—with high valence rating (mean 7.00 and SD ± 0.61) and high arousal (mean 4.61 and SD ±0.80), *unpleasant* (33 objects)—with low valence rating (3.17 ± 0.60) and high arousal (4.78 ± 0.73), or *neutral* (48 objects)—with valence rating around 5 (5.33 ± 0.27) and low arousal (2.38 ± 0.35). Table 1 describes the valence, arousal, and emotional category for each object. Instead of strictly selecting or controlling objects to have the same or very similar shapes, sizes, weight and other features - which would most likely be noticed by participants and thus interfere with their understanding of the task or their judgments of affordance (i.e., if all objects were very similar, subjects may become desensitized, uninterested or confused)—we preferred to have a more naturalistic, relevant and easily recognizable set of stimuli. Thus, the set of objects include, for example: food (in various presentations such as appetizing, rotten, infested with flies’ worms or inside package), jewelry, money, cosmetics, office supplies, embalmed or dissected animals or their parts, toys, children-related objects or clothing and electronic devices/gadgets (see Table 1 for a complete list).

**Table 1:** Description of objects used as stimuli.

Emotional category	Object	Valence	Arousal	Adjusted size (cm)
Neutral	Acetone Glass	5.43	2.89	4.00
Neutral	Surgical tape	5.02	2.24	4.00
Neutral	Porcelain apple	5.67	2.49	5.00
Neutral	Battery	5.11	2.46	2.05
Neutral	Braces box	4.43	2.38	5.10
Neutral	Calculator (slab-shaped)	5.02	2.90	4.00
Neutral	Calculator (stick-shaped)	5.33	2.19	2.30
Neutral	Pen box	5.65	2.13	2.30
Neutral	Sunglasses box 1	5.17	1.97	6.25
Neutral	Paper clips box	5.24	1.98	4.50
Neutral	Perfume bottle	5.81	2.70	1.70
Neutral	Floss	5.73	2.41	3.30
Neutral	Flower shaped candle	5.41	2.37	5.25
Neutral	Garage controller	5.49	2.90	3.50
Neutral	Glue	5.44	2.24	3.40
Neutral	Golden bracelet	5.22	2.03	6.75
Neutral	Golden watch	5.59	2.67	2.80
Neutral	Grater	4.90	2.94	3.45
Neutral	Green scissors	5.24	2.24	3.00
Neutral	Guava	5.59	2.29	5.00
Neutral	Hair brush	5.46	2.52	5.10
Neutral	Highlighter	5.63	2.73	5.50
Neutral	Jaw	5.14	3.13	6.50
Neutral	Kama Sutra box	5.29	2.78	7.00
Neutral	Fluorescent lamp	5.38	2.32	6.15
Neutral	Money bag	5.46	2.30	4.75
Neutral	Nail polish	5.68	2.56	3.00
Neutral	Nail polish box	5.48	2.32	4.50
Neutral	Onion cut in half	5.08	3.08	4.50
Neutral	Plug adapter	5.19	2.35	5.15
Neutral	Plum	5.87	2.48	5.00
Neutral	Pointer	5.17	2.00	2.00
Neutral	Porcelain tomato	5.54	2.89	6.75
Neutral	Sunglasses box 2	5.25	1.73	6.25
Neutral	Rounded purple box	5.29	2.00	3.75
Neutral	Razor blade	5.19	2.98	1.40
Neutral	Roll	5.02	1.76	4.20
Neutral	Round mirror	5.21	2.21	4.30
Neutral	Screwdriver	5.32	2.27	1.75
Neutral	Silver bracelet	5.56	2.37	5.75
Neutral	Soap dish	5.17	2.11	7.85
Neutral	Square mirror	5.33	2.03	4.55
Neutral	Stapler	5.24	2.21	3.05
Neutral	Swab bundle	5.60	2.14	4.25
Neutral	Toilet paper roll	5.05	2.10	4.50
Neutral	Tooth brush	5.52	2.63	1.50
Neutral	Adhesive tape	5.17	1.81	4.40
Neutral	Yellow cube	5.08	2.17	5.50
Pleasant	Baby sandal	6.40	3.76	7.00
Pleasant	Cake with chocolate icing	6.60	4.92	4.00
Pleasant	Car keys	6.56	4.27	2.75
Pleasant	Brigadeiro - Brazilian chocolate candy	8.05	5.56	3.50
Pleasant	Stack of condoms	6.62	5.14	4.25
Pleasant	Closed pack of condoms	6.65	4.76	4.50
Pleasant	Credit card	6.46	4.52	3.15
Pleasant	Gift box	7.56	5.29	6.30
Pleasant	iPod	6.71	4.03	3.05
Pleasant	Jewel box	6.35	3.33	4.60
Pleasant	Lemon	6.35	3.37	5.20
Pleasant	Beijinho – milky Brazilian candy	7.27	5.19	3.85
Pleasant	Money roll	8.10	6.08	3.80
Pleasant	Prestígio – coconut and chocolate bar	7.13	5.24	3.35
Pleasant	Soap box	6.57	3.49	4.70
Pleasant	Sweet bread	7.10	4.60	4.25
Pleasant	Talento – chocolate bar	7.76	5.25	5.20
Pleasant	Teddy bear	6.60	3.51	8.00
Pleasant	Bread toast	7.38	4.65	3.00
Pleasant	Twix – chocolate bar	8.14	5.54	3.35
Pleasant	Wedding souvenir	6.52	4.25	5.00
Unpleasant	Beetle	3.71	5.02	4.25
Unpleasant	Braces	3.86	3.56	2.60
Unpleasant	Bread with flies	2.35	5.10	4.25
Unpleasant	Brush with hair	2.41	5.02	5.10
Unpleasant	Cactus	4.70	3.71	3.00
Unpleasant	Cake with hair	2.21	5.71	4.00
Unpleasant	Chewing gum with hair	2.70	5.08	2.00
Unpleasant	Chicken foot	2.90	4.57	2.75
Unpleasant	Chicken head	2.54	5.29	4.50
Unpleasant	Cockroach	2.95	4.95	4.30
Unpleasant	Dirty menstrual pad	2.54	5.00	3.75
Unpleasant	Dirty steel wool	3.21	3.90	2.50
Unpleasant	Embalmed human foot	4.05	4.40	4.75
Unpleasant	Embalmed human heart	4.25	4.05	5.75
Unpleasant	Embalmed snake 2	3.70	5.79	1.50
Unpleasant	Embalmed moth caterpillar	3.10	4.87	2.25
Unpleasant	Embalmed scorpion	3.25	5.00	1.00
Unpleasant	Embalmed spider	3.24	6.29	3.00
Unpleasant	Embalmed centipede	2.97	5.46	0.75
Unpleasant	Embalmed caterpillar	3.33	4.21	1.75
Unpleasant	Half of a fetal head	2.65	5.49	6.00
Unpleasant	Fish head	3.54	4.44	6.50
Unpleasant	Gecko	3.63	5.29	1.25
Unpleasant	Gizzard	3.14	4.59	5.60
Unpleasant	Guava with worms	2.52	5.16	3.75
Unpleasant	Lizard	3.51	5.02	2.00
Unpleasant	Mouse trap	4.10	4.03	3.65
Unpleasant	Open mouse	2.41	5.97	2.50
Unpleasant	Rotten banana	3.30	3.40	2.25
Unpleasant	Rotten mango	2.84	4.17	4.50
Unpleasant	Rotten potato	3.08	3.48	4.00
Unpleasant	Embalmed snake 1	2.90	4.89	2.50
Unpleasant	Water bug	3.16	4.94	3.75

### 2.3 Object size

As alluded to earlier, plenty of studies have shown and described the influence of object features on grasping movements. Usually, studies on this topic employ symmetrical or regular shaped objects - such as spheres, cubes and specially cylinders of varying ratios between its dimensions - to evaluate how object size, position, orientation, and other variables influence the kinematics of reaching and grasping movements. Though this approach offers easy control over experimental conditions, most objects people interact with daily are considerably irregular, especially those typically considered as pleasant or unpleasant. Also, the term “object size” is, obviously, a reductionism for the sake of simplicity, since objects have multiple dimensions that could, presumably, influence movement parameters simultaneously and in variable ways. Nevertheless, when grasping irregular objects, the final grip aperture is inevitably determined by the contact locations on the object’s surface (Goodale et al., 1994). Evidently, determining these locations from visual information is essential for movement planning, and indeed has been found to be highly dependent on object shape for asymmetrical objects (Cuijpers et al., 2004). Therefore, when looking at an object and judging the appropriate manipulation (the task in our study), the distance between the perceived grasping locations on its surface is presumably the most relevant measure concerning object geometry (Smeets and Brenner, 1999), whereas other perceived dimensions of the object may not be relevant at all (Smeets et al., 2002). This is especially plausible considering the nature of our task, which, as we will explain ahead, restricted the manipulation possibilities to only two types of grasping that have similar kinematic profiles and are motorically controlled based on similar object parameters. The issue is then knowing for each object what are the grasping locations used for these motor computations.

Cuijpers et al. (2004) have found that when asked to use a precision grip to pick up elliptical cylinders of varying aspect ratios, orientations and positions, participants consistently tend to grasp these objects along their minor/thinner axis (68% of their trials), because this attenuates the effects of errors in aligning the hand grip axis with the chosen object axis, reducing the chance of instability compared to choosing a greater axis. As expected, the maximum grip aperture during the reach movement was strongly predicted by the selected axis’ length, but it was also influenced by the other horizontal axis when it was bigger than the axis selected for grasping. Based on these results and the previous notions, we reasoned that these two smaller axes may account for most of the effects that object dimensions have on affordance judgment in our experiment setting, given their key role in motor control of actual grasping movements. Therefore, our approach for dealing with the complexity introduced by the heterogeneity in object geometry was to calculate the arithmetic mean of the two smaller axes for each object and use this single value (herein referred as “*Adjusted size*”) as a proxy for the influence that object dimensions have on affordance judgment. Thus, each object had its dimensions (height, width, and length) measured and was attributed an Adjusted size (arithmetic mean between its two smaller dimensions). Table 1 describes the dimensions and adjusted size of each object. Note that these measurements were made with the real objects, and not with the pictures we subsequently took of them and used as stimuli in the experiment.

### 2.4 Procedure

**Figure 1:**
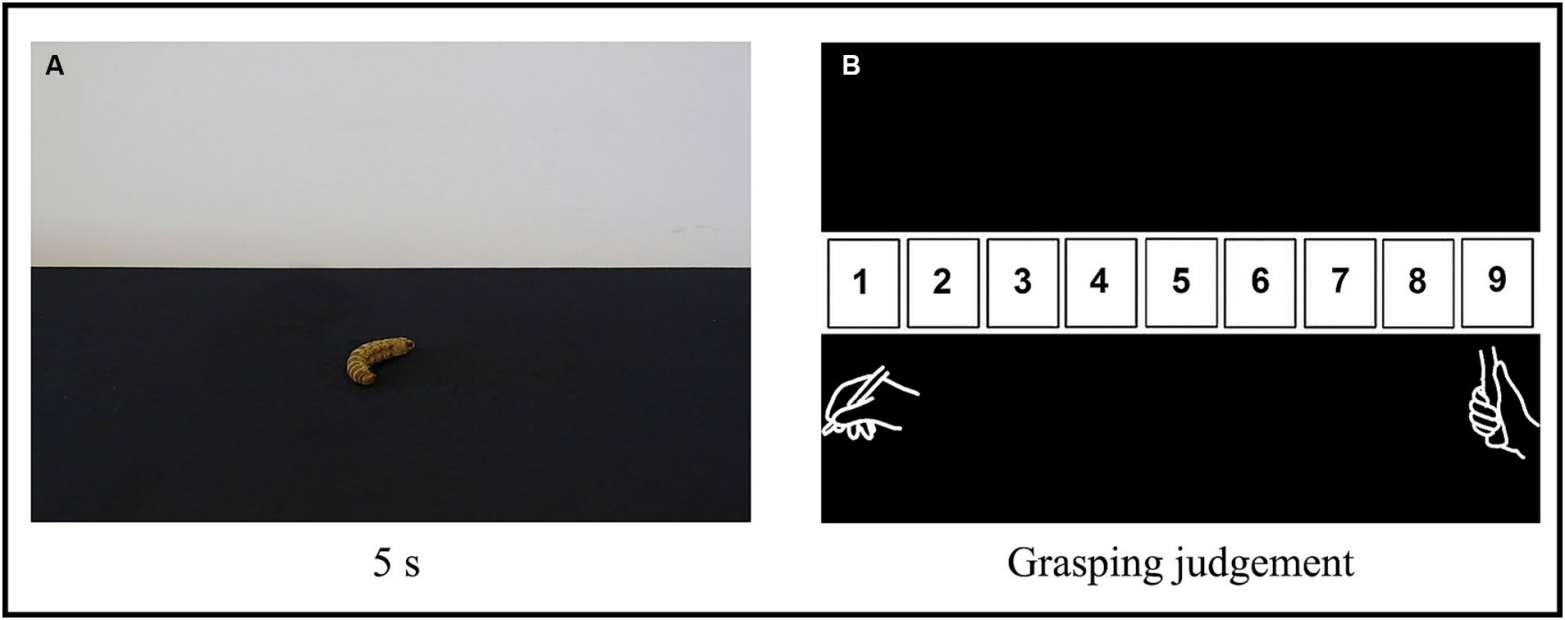
Experimental procedure. **(A)** Example of an object (an embalmed caterpillar). Each image was presented for 5 s, in random order, and participants were instructed to passively but attentively observe each one. **(B)** The custom scale of grasping judgment that participants used to classify the objects based on the type of grip they afforded (i.e., attribute an “affordance score”). The number 1 corresponds to the greatest suitability or appropriateness for the object to be manipulated by a precision grip and 9 by a power grip. Participants were instructed to click with a computer mouse on the number of the scale that best corresponded to how they judged each observed object should be manipulated, considering two types of grasping: *precision grip* (depicted below the number 1) and *power grip* (depicted below the number 9).

Participants sat on a comfortable chair positioned in front of a computer screen (1280×800 pixels, 19-inch, refresh rate of 75 Hz, at 70 cm from the participant) and a mouse stand in a standard position in a brightly lit room. The task consisted in a classification of objects according to the type of grip they afforded (i.e., affordance judgment) by means of a custom numeric scale, which ranged from 1 to 9 (Figure 1), in which 1 corresponded to the greatest suitability or appropriateness of the observed object to be manipulated by a precision grip and 9 by a power grip.

Participants were instructed to attentively observe each picture and, after it faded away, click with a computer mouse on the number of the scale that best corresponded to how they judged the observed object should be manipulated. They should consider two prototypes of grasping: a precision grip, in which there is a predominance of the opposition between the thumb and index-finger, and a power grip, in which the closure of the whole hand and all fingers is predominant (Castiello, 2005; Jarque-Bou et al., 2016). Therefore, for example, if a participant judges an object to be best manipulated by a precision grip rather than a power grip, they should give it an “affordance score” closer to 1.

At the beginning of the experiment, the task was explained, and a training session was run with four additional objects that were only used for this training (not included in the data recording session or data analysis). Participants were then asked to verbally explain back to the experimenter what they had to do and what the numbers on the scale meant, all to ensure they understood the task. Lastly, they were instructed to not speak with the experimenter during the session and to complete the whole trial uninterrupted in one sitting (which lasted about 20 min). When participants declared to be ready, the trial began: pictures of each of the 102 objects were presented one at a time, in random order, for 5 s, each followed by the numerical scale (Figure 1). Only after the participant clicked on their chosen number, was the next picture presented. Participants were not asked to perform this task as fast as they could, but rather to choose the number that best represented their judgment as previously explained. The experiment was controlled by a program we developed in Matlab software (MathWorks).

### 2.5 Data analysis

We used JASP Version 0.14.1 (JASP Team, 2024) to perform a linear mixed effects analysis on the relationship between affordance scores (dependent variable) and the emotional category (predictor variable of interest) and Adjusted size of objects (covariate), entering them and their interaction term into the model as fixed effects. As random effects, we had by-object and by-participant (grouping factors) random intercepts for all fixed effects terms and by-participant random slopes for the emotional category only, because this was the most complex random effects structure that allowed for a non-singular fit and because by-object random slopes were non-applicable (since each object always had the same value for both Adjusted size and emotional category). The model was fitted using restricted maximum likelihood and type II Sum of Squares (given the imbalance in number of observations between emotional categories). We tested for main effects and interactions by the Kenward-Roger method (Kenward and Roger, 1997). Significant effects (⍺ = 0.05) were followed up with post-hoc tests using Bonferroni correction of *p*-values.

## 3 Results

**Figure 2:**
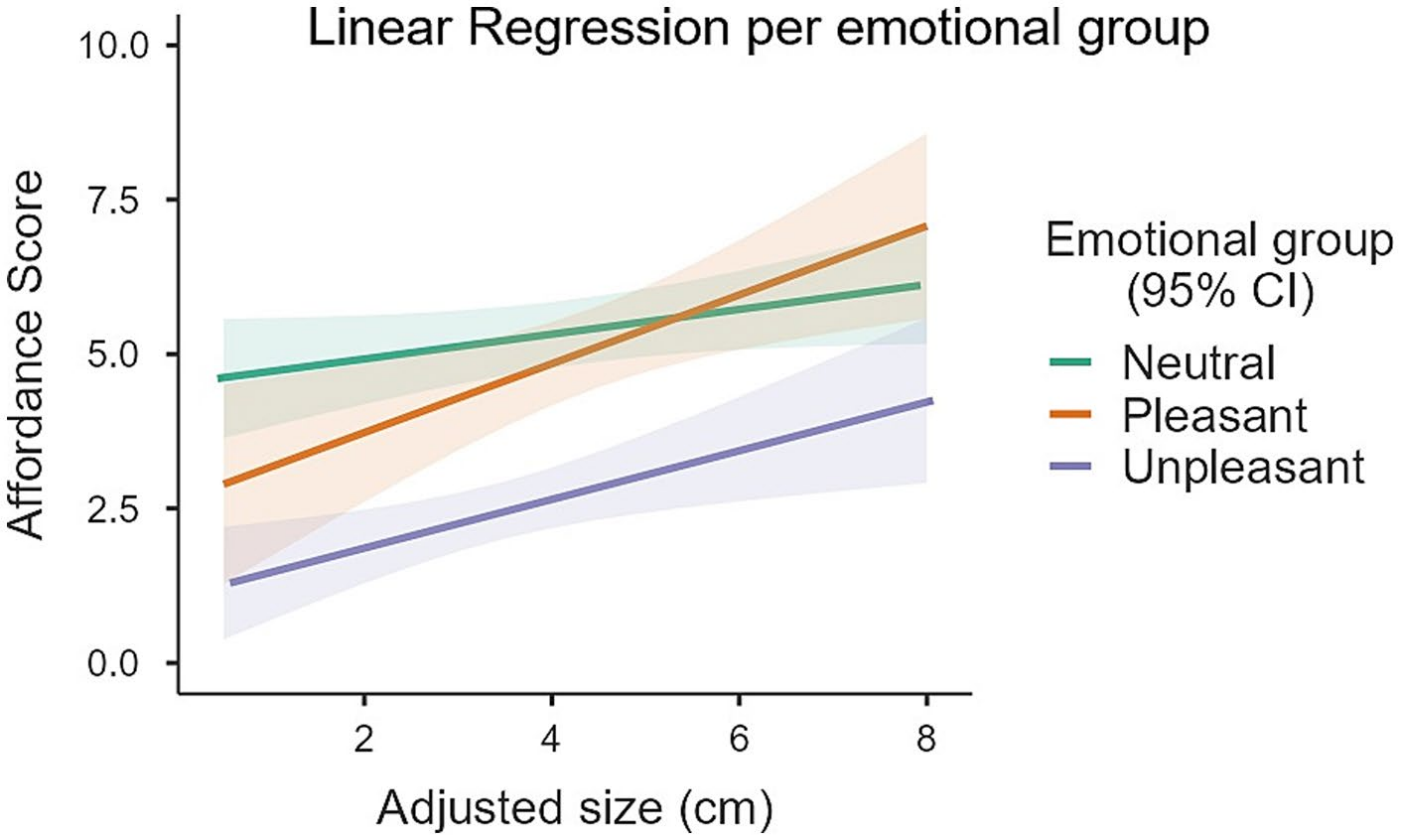
Linear regressions between Affordance scores and Adjusted size for each Emotional group of objects. Unpleasant objects scored lower (indicating higher preference for precision grip) than the neutral and pleasant objects (*p < 0.001), which themselves did not differ significantly. A main effect of Adjusted size was also found: bigger objects afforded a higher preference for power grasp (*p* < 0.001). No significant interaction was found between Adjusted size and Emotional group.

Significant main effects of both emotional category [*F*_(2, 108.76)_ = 10.20, *p* < 0.001] and Adjusted size [*F*_(1, 96)_ = 17.22, *p* < 0.001] were found. The interaction between emotional category and Adjusted size was not significant [*F*_(2, 96)_ = 1.54, *p* = 0.22]. Post-hoc tests indicated that the model coefficient for the Adjusted size term was 0.38 points [SE = 0.09, *t*_(96)_ = 4.46, *p* < 0.001]. This means that for each 1 cm increased in the Adjusted size of an object the mean affordance score would increase by an estimated 0.38 points (an increase in affordance scores indicates a higher preference for a power grip). As an example, for an increase of 1 standard deviation in Adjusted size - 1.55 cm in our sample of objects - the corresponding increase in affordance scores would be of 0.60. Post-hoc tests revealed that, considering an Adjusted size of 4.03 cm (the mean across all objects), unpleasant objects (2.69 ± 0.25) received lower affordance scores (indicating higher preference for a precision grip) than both neutral (5.32 ± 0.27, *t* = 6.71, *p* < 0.001) and pleasant (4.86 ± 0.34, *t* = 4.84, *p* < 0.001) objects. The difference in affordance scores between neutral and pleasant objects was not significant (*t* = 1.37, *p* = 0.53). Figure 2 summarizes these results.

*Post-hoc* tests revealed that unpleasant objects received lower affordance scores (indicating higher preference for a precision grip) than both neutral (*t* = 6.71, *p* < 0.001) and pleasant (*t* = 4.84, *p* < 0.001) objects. Because affordance scores were also simultaneously influenced by Adjusted size (as described above) and object Adjusted sizes were not homogenous between groups, a simple group-level comparison between emotional categories would be misleading. Instead, what is more informative and appropriate is to make these comparisons by first setting a level or baseline value for the Adjusted size and then compare estimated marginal means between groups. So, if we consider hypothetical objects with an Adjusted size of, for example, 4.03 cm (the mean across all 102 objects), estimated affordance scores were 2.69 ± 0.25 for unpleasant objects, 5.32 ± 0.27 for neutral objects and 4.86 ± 0.34 for pleasant objects. Table 2 provides this and two other examples of Adjusted sizes for such comparisons: 4.03 cm ± 1.55 cm (mean ± the standard deviation of Adjusted sizes across all objects).

**Table 2:** Comparison between emotional categories using estimated marginal means.

Adjusted size (cm)	Emotional category	Affordance score estimate	SE	95% CI Lower boundary	95% CI upper boundary
2.483	Neutral	5.004	0.330	4.357	5.650
	Pleasant	3.998	0.496	3.026	4.969
	Unpleasant	2.075	0.264	1.557	2.593
4.033	Neutral	5.315	0.267	4.791	5.838
	Pleasant	4.862	0.339	4.197	5.526
	Unpleasant	2.686	0.249	2.198	3.174
5.583	Neutral	5.626	0.299	5.040	6.212
	Pleasant	5.725	0.399	4.944	6.506
	Unpleasant	3.297	0.380	2.552	4.042

## 4 Discussion

Our main finding is that participants considered unpleasant objects more fitting for manipulation by a precision grip rather than a power grip when compared to neutral and pleasant objects, which themselves did not differ in this affordance judgment. The data also shows that bigger objects (i.e., bigger Adjusted size) increased the preference for a power grasp., regardless of their emotional category (i.e., no interaction between these factors): 1 cm increase in the Adjusted size of an object would increase the mean affordance score by an estimated 0.38 points. Comparing the magnitude of these two effects, the influence of emotion on affordance judgment is quite considerable: on average, unpleasant objects received an affordance score 2.63 and 2.17 points lower than neutral and pleasant objects, respectively. This would be equivalent to the effect of a difference in Adjusted size between two objects of 6.92 cm and 7.71 cm, respectively. Consider that the difference in Adjusted size between our smallest and biggest objects (Embalmed centipede—0.75 cm and Teddy bear—8.0 cm) is of 7.25 cm. In other words, comparing neutral or pleasant objects to unpleasant objects yields differences in affordance judgment of similar magnitude to comparisons between the smallest and biggest objects in our set of stimuli.

Affordance has been a critical concept in our study because it serves as a paradigm in understanding *object-directed action planning*. For this process the individual must assess, among other things, relational properties of the object to be interacted with, such as size, orientation and distance, as well as action goals (Jeannerod et al., 1995; Paulignan et al., 1997; Cattaneo et al., 2005; Riehle, 2005; Andersen and Cui, 2009; Marangon et al., 2011). Our study asks whether the emotional value of the object (or attributed to it) is one such factor influencing action planning. As many studies have shown, viewing emotional stimuli leads to certain modulations in the motor system, such as in postural control (Azevedo et al., 2005; Facchinetti et al., 2006), force production (Coombes et al., 2006), arm and eye kinematic profiles (Ferri et al., 2010; West et al., 2011; Esteves et al., 2016), corticospinal excitability (Oliveri et al., 2003; Coombes et al., 2007a,b, 2009; Coelho et al., 2010; Nogueira-Campos et al., 2014, 2016) and electroencephalographic activity (De Oliveira et al., 2012), so it was reasonable to hypothesize that it would also affect affordance judgment.

A representational account of object-directed action planning (e.g., Cisek, 2007) would typically put a heavy emphasis on how the brain processes certain aspects of visual stimuli and integrates it with endogenous information (like action goals) to form visual or motor representations of objects, their properties or action possibilities. From here, the critical step would be to explain how the brain performs *action selection*: from a “motor repertoire” of many possibilities, how do we get to one *specific* action that should be planned, executed, perceived or understood as appropriate. In this framework, our findings support the idea that emotion is an important component (much like object size) that the visuo-motor system takes into account during action planning.

In contrast, the ecological psychology approach emphasizes that such *specification* of actions is already heavily determined by the environment, and therefore may depend to a much lesser extent on internal processes in the brain. For example, Gibson (1979) points out that light travels through, is absorbed and reflected by the environment in very different and specific ways according to the surfaces, properties and dispositions of objects. Therefore, the light that reaches the retina is already heavily organized or structured in arrays that are informative of the environment. These ambient arrays are also further structured and changed by characteristics and actions of the observer, therefore also being informative of the relationships between animal and environment. Gibson then proposes that animals can perceive some of these structural properties or variables of light arrays to directly perceive specific action possibilities (affordances). When used to understand action planning, this framework shifts some of the “explanatory load” from the brain to the environment. Our findings suggest that the perception (or at least judgment) of affordances is influenced by the emotional value of objects. This has certain theoretical implications for affordances that we will return to ahead.

One expectation that one could have for this experiment was that, because emotions affect visual perception and affordance judgment depends on visual features of objects, we would, therefore, find that affordance judgments reflect this same emotional effect on visual perception. Specifically, as we mentioned in the Introduction, unpleasant stimuli can be perceived as bigger in certain situations (such as in Van Ulzen et al., 2008; Leibovich et al., 2016), especially in threatening contexts. This could imply that we should also have found this size overestimation reflected in our data, in the form of higher affordance scores for unpleasant objects (when in fact we found the opposite). That is, if unpleasant objects are perceived as being bigger, then subjects would rate them as being more fitting for power grasping (and give them a higher affordance score).

Indeed, in a series of experiments, Sun et al. (2021) asked subjects to visually estimate the size and distance of small rectangular wooden plaques placed on top of either wooden blocks (neutral condition), rat toys (unpleasant) or squirrel toys (pleasant). In another experiment, subjects had to reach and grasp these wooden plaques in similar emotional conditions induced by two above mentioned objects while kinematic recordings were made. In accordance with the literature on emotional effects on visual perception, the wooden plaques were perceived to be bigger and closer when placed on top of unpleasant stimuli. However, analogous to our results, when reaching to grasp these objects, subjects performed a smaller grip aperture in the unpleasant condition compared to neutral and pleasant (though, in our case, the source of emotion and target of affordance judgments are the same). This effect was present when subjects could not see their hands and the target object but was absent when subjects had proper visual feedback. As mentioned earlier, maximum grip aperture during reaching is a kinematic parameter commonly reported to correlate with the real size of the grasped object, so this “overestimation of size but smaller aperture when grasping” for unpleasant stimuli seems puzzling. When taken together, our experiment and the literature presented thus far suggests that (1) subjects are overestimating the size of unpleasant stimuli, (2) they are indeed judging their affordance to be of a finer or smaller grip and (3) this effect is even affecting the initial motor planning of grasping actions, but (4) it can be “corrected” by the online adjustments during movement performance. Additionally, our results suggest that the expected relation between object size and grasping affordance is actually present in these situations, but it is being outmatched by the effect of emotion on the outcomes measured, at least in the initial processes.

From a representational perspective, affordance selection is a multi-factorial process depending on inputs from and interactions between multiple brain systems (see Thill et al., 2013 for an overview). Even if our emotion-laden objects had their size under/overestimated to some extent, this alone might not have been sufficient to change grasping judgments. There are other important components of affordance processing that unpleasantness could also have affected, such as high-level goals. Indeed, there is evidence that movement selection and the kinematics of grasping actions are sensitive to changes in determinants of action goals that are prone to emotional influence, such as social context and interaction and emotional feedback from others (Becchio et al., 2008, 2010; Ferri et al., 2010, 2011). Thus, it is reasonable to think that the object’s emotional value might influence affordance selection through ways other than changes in size perception, especially in scenarios where interaction with the observed object is heavily implied. For example: in our experiment the unpleasantness might have conveyed the need for a more precise grasp that favored careful manipulation and minimized physical contact with an aversive stimulus. This could have outweighed any effects that size overestimation would have had in defining a more behaviorally appropriate interaction with these objects. In summary, even if subjects overestimated the size of unpleasant objects, that was not the whole task asked of them: the motor visuo-system needs to consider other information, such as emotional valence, when judging affordances.

Finally, maybe the most important implication of this experiment is to reconsider what affordances are if their judgment can be influenced by emotions. If affordances are what the environment affords to the observer and unpleasant objects afford a precision grasp in part *because* they “are” unpleasant, then is this unpleasantness part of what constitutes (or specifies) the object’s affordance?

Certainly, we would consider relative size, orientation, shape, distance and other *relational features of the animal-environment system* to be “part of what constitutes (or specifies) the object’s affordance.” They determine, limit or influence how the animal can or should interact with the environment. They also determine how the observer *judges* these action possibilities (Warren, 1984; Warren and Whang, 1987). Likewise, our experiment shows that emotional valence also determines how observers judge action possibilities. And emotions are also “relational,” in the sense that a complete account of emotional phenomenon involving objects will depend on both the specific nature of the object and animal, as well as their relationships; much like a description of relative sizes, shapes or other “typical” affordance determinants. Therefore, it seems that affective aspects of our experience of objects both function and can be described in very similar terms as to how we analyze and theorize about affordances; or at least they have some considerable conceptual overlapping worth exploring. The central question we are raising here is not just whether emotions influence affordance-related phenomena (like graspability judgments), but whether they constitute or overlap with what affordance perception is.

A distinction one could make to maybe “separate” affordances and emotions in the context of this experiment is between *possible* and *appropriate* movements. Affordances could then be only descriptions of (many) *possible* actions but when subjects are asked to judge how objects *should* be manipulated, they are judging which of these possibilities is the most *appropriate* one. In this way, the affective value of the object could be influencing only this judgment task, and not what the affordances are or their perception. Indeed, Gibson took this sort of stance on this issue (Reed and Jones, 2019):

*“The affordances of the environment are permanent, although they do refer to animals and are species-specific. The positive and negative valences of things that change when the internal state of the observer changes are temporary. The perception of what something affords should not be confused with the ‘coloring’ of experience by needs and motives. Tastes and preferences fluctuate. Something that looks good today may look bad tomorrow but what it actually offers the observer will be the same.”*

The problem is whether we can meaningfully and practically distinguish between “the perception of what something affords” and our “colored experience” of, about or involving that thing. Maybe this distinction is possible if we reduce affordances to be some abstract, mechanical and experience-independent description of “what actions are physically possible.” But as we expressed in the introduction, much of the conceptual utility of affordances stems from this phenomenological aspect of objects (or the environment) inviting, soliciting, demanding or evoking certain *appropriate* interactions. If we still wish to conceptualize affordances in a way that excludes this aspect, then affordances would just be “what something affords” in a very broad sense. We would still need to declare and explain how animals seem to perceive, experience or react to *specific* or *appropriate* affordances, especially if we also wish to argue that emotions affect perception.

Maybe in a seemingly “emotionally neutral” situation, like when judging whether we can climb stairs of different sizes (Warren, 1984), the proposition that animals can directly perceive affordances seems reasonable and sufficient to explain this “climbability” perception and even related behavior. It does not seem necessary to reference affective phenomena to explain such judgments because stairs of different riser heights do not seem to have some obvious and different affective value assigned to them. But what happens when the object does have some rather obvious emotional value relevant for motor behavior? If the object’s affordance does not reference this emotional value, then perceiving affordances is not enough to explain affordance judgments, much less visually guided behavior.

## 5 Conclusion

Our data supports the hypothesis that affordance judgment is influenced by the emotional value of objects, which specifically favors affordances compatible with careful manipulation and minimal physical contact with aversive stimuli. The magnitude of this phenomenon was considerable when compared to the effect of object size on affordance judgment, which was also present as expected. These effects did not interact: emotional influences were constant for both small and big objects and were not selectively affected by or restricted to a certain range of object sizes. In summary, this experiment shows that the emotional value we attribute to objects is an important factor on motor control: it influences our understanding of how we should interact with objects, alongside other factors such as object size. Finally, this experiment suggests that affordance judgments are influenced by emotional value, which merits a more thorough, dedicated theoretical work exploring the ontological relationship between affective phenomena and affordances.

## Data availability statement

The original contributions presented in the study are included in the article/Supplementary material, further inquiries can be directed to the corresponding author.

## Ethics statement

The studies involving humans were approved by Comitê de Ética em Pesquisa com Seres Humanos—Universidade Federal de Juiz de Fora (UFJF). The studies were conducted in accordance with the local legislation and institutional requirements. The participants provided their written informed consent to participate in this study.

## Author contributions

MF: Conceptualization, Formal analysis, Investigation, Methodology, Writing – original draft. IA: Conceptualization, Formal analysis, Investigation, Methodology, Visualization, Writing – original draft. GS: Conceptualization, Writing – review & editing. LK: Software, Writing – review & editing. AN-C: Conceptualization, Formal analysis, Methodology, Supervision, Writing – review & editing.

## Footnotes

^1^ https://osf.io/zstmy/
